# Peripheral Precocious Puberty as the Initial Manifestation of Juvenile Granulosa Cell Tumor in Early Childhood

**DOI:** 10.7759/cureus.116834

**Published:** 2026-09-24

**Authors:** Sanaa Bouramdane, Kenza Elmkaddem, Nawal Hammas, Meryem Boubbou, Khalid Khattala, Youssef Bouabdallah, Sarra Benmiloud

**Affiliations:** 1 Department of Pediatric Hematology and Oncology, Hassan II University Hospital, Faculty of Medicine, Pharmacy and Dental Medicine, Sidi Mohamed Ben Abdellah University, Fez, MAR; 2 Department of Pathology, Hassan II University Hospital, Faculty of Medicine, Pharmacy and Dental Medicine, Sidi Mohamed Ben Abdellah University, Fez, MAR; 3 Department of Mother and Child Radiology, Hassan II University Hospital, Faculty of Medicine, Pharmacy and Dental Medicine, Sidi Mohamed Ben Abdellah University, Fez, MAR; 4 Department of Pediatric Surgery, Hassan II University Hospital, Faculty of Medicine, Pharmacy and Dental Medicine, Sidi Mohamed Ben Abdellah University, Fez, MAR

**Keywords:** fertility-sparing surgery, inhibin b, juvenile granulosa cell tumor, pediatric ovarian tumor, peripheral precocious puberty, sex cord–stromal tumor

## Abstract

Juvenile granulosa cell tumor (JGCT) is a rare ovarian sex cord-stromal tumor (SCST) occurring predominantly in children and adolescents. It is characterized by estrogen secretion, which often leads to peripheral precocious puberty and allows early clinical detection despite the tumor's rarity. We report the case of a previously healthy two-year-nine-month-old girl who presented with progressive breast development, persistent leukorrhea, and vaginal bleeding. Hormonal evaluation revealed suppressed luteinizing hormone and follicle-stimulating hormone concentrations associated with a markedly elevated inhibin B level (>5000 pg/mL), consistent with a hormonally active ovarian SCST. Contrast-enhanced computed tomography and pelvic magnetic resonance imaging demonstrated a large complex left ovarian mass with imaging features suggestive of malignancy. The patient underwent fertility-sparing left oophorectomy with comprehensive surgical staging. Histopathological examination and immunohistochemistry confirmed the diagnosis of JGCT, and the disease was classified as International Federation of Gynecology and Obstetrics stage IA. No adjuvant chemotherapy was administered, and the patient was managed with active surveillance. Hormonal abnormalities normalized within two months after surgery, and no evidence of recurrence was observed after 12 months of follow-up. This case highlights the importance of considering an estrogen-secreting ovarian tumor in very young girls presenting with peripheral precocious puberty. Early recognition of endocrine manifestations, combined with appropriate imaging, hormonal evaluation, and fertility-preserving surgery, enables excellent outcomes in localized disease. Inhibin B also represents a valuable individualized biomarker for postoperative surveillance when elevated at diagnosis.

## Introduction

Juvenile granulosa cell tumor (JGCT) is a rare ovarian sex cord-stromal tumor (SCST), with an estimated annual incidence as low as two cases per million children and accounting for approximately 15% of pediatric ovarian neoplasms [1]. Its clinical significance is largely related to its hormone-secreting activity, particularly autonomous estrogen production [2]. In very young girls, this endocrine activity may manifest as breast development, vaginal discharge, or vaginal bleeding before an ovarian mass becomes clinically apparent [1]. Such manifestations reflect peripheral, gonadotropin-independent precocious puberty, which should be distinguished from central precocious puberty resulting from premature activation of the hypothalamic-pituitary-gonadal axis [2]. Because these endocrine manifestations may precede abdominal symptoms, they can provide an important early clue to the presence of an underlying ovarian tumor [3]. When diagnosed at a localized stage, JGCT generally carries an excellent prognosis following complete surgical resection [4].

We report the case of a two-year-nine-month-old girl with an ovarian JGCT revealed by progressive estrogenic manifestations evolving over five months before the detection of a large pelvic mass. This case highlights the importance of recognizing progressive peripheral precocious puberty as an early diagnostic clue to a hormone-secreting ovarian tumor and provides an opportunity to discuss the clinical, hormonal, radiological, pathological, therapeutic, and prognostic features of pediatric JGCT in light of recent evidence.

## Case presentation

A previously healthy two-year-nine-month-old girl with no relevant personal or family history was referred to our department after surgical resection of a left ovarian mass. Five months before surgery, her parents had noticed unilateral left breast enlargement, which gradually became bilateral. Breast development was painless, with no areolar pigmentation, and was accompanied by persistent scant whitish vaginal discharge from the onset of symptoms. Fifteen days before her initial presentation, she developed several episodes of mild vaginal bleeding, together with the progressive appearance of a left pelvic mass.

At initial presentation, the patient was in good general condition, with normal vital signs for her age. She weighed 16 kg (−1 SD) and measured 96 cm in height (mean for age). Pubertal examination revealed bilateral breast development (Tanner stage B2) and a few long pubic hairs confined to the labia majora, without other signs of virilization. Abdominal examination revealed a firm, fixed, well-defined, non-tender left lateropelvic mass measuring approximately 10 cm in its greatest diameter. The remainder of the physical examination was unremarkable.

During the initial preoperative evaluation, hormonal assessment demonstrated suppressed gonadotropin levels, with luteinizing hormone (LH) <0.10 IU/L (reference range, <1 IU/L) and follicle-stimulating hormone (FSH) <0.30 IU/L (reference range, <11 IU/L), associated with a markedly elevated inhibin B concentration (>5000 pg/mL; reference range, <182 pg/mL). Serum anti-Müllerian hormone (6.88 ng/mL; reference range, 0.2-7.3 ng/mL), dehydroepiandrosterone sulfate (4.2 ng/mL; reference range, 1.3-9.8 ng/mL), and androstenedione (<0.24 ng/mL; reference range, <0.34 ng/mL) were within normal limits. Serum alpha-fetoprotein (AFP) (1.3 ng/mL; reference range, <8.78 ng/mL) and β-human chorionic gonadotropin (β-hCG) (<0.10 mIU/mL; reference range, <5 mIU/mL) were also within normal limits. Serum estradiol was requested as part of the initial hormonal evaluation; however, the result was unavailable for review. Bone age assessment was not performed during the initial preoperative evaluation.

Contrast-enhanced abdominopelvic computed tomography (CT) revealed a large, heterogeneous, lobulated, enhancing midline suprauterine mass, probably arising from the left ovary and measuring 103 × 63 × 117 mm. The lesion was closely related to the uterus, sigmoid colon, upper rectum, and adjacent ileal loops, with focal loss of intervening fat planes, initially raising concern for local extension. Bilateral dilatation and tortuosity of the laterouterine veins, more pronounced on the left, were also observed. The uterus was mildly enlarged, with endometrial thickening to 5 mm, and moderate intraperitoneal ascites was present (Figure 1).

**Figure 1:**
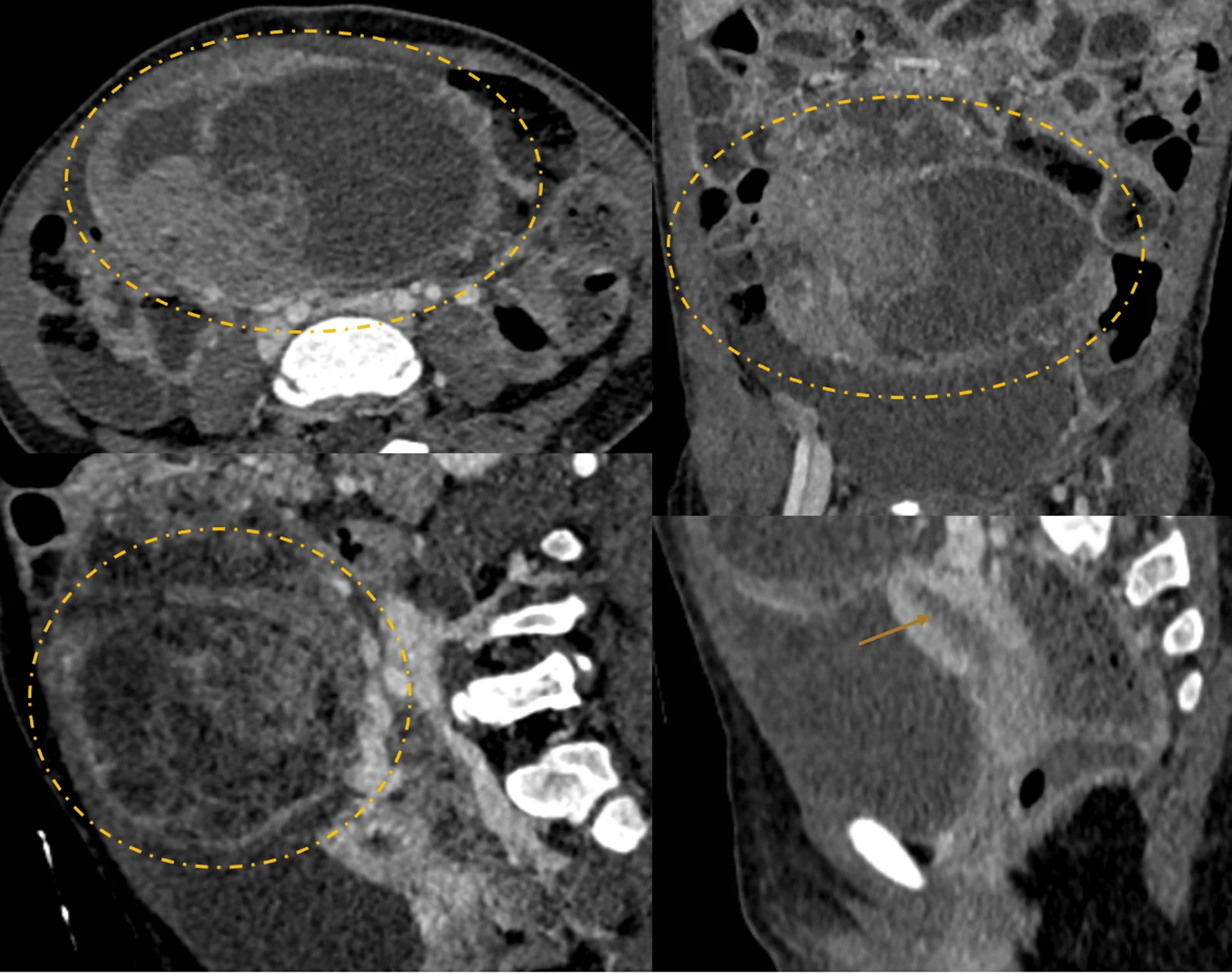
Contrast-enhanced abdominopelvic computed tomography Contrast-enhanced abdominopelvic computed tomography showing a left ovarian solid-cystic mass with heterogeneous enhancement (yellow circle), associated with endometrial thickening (orange arrow).

Pelvic magnetic resonance imaging (MRI) confirmed a solid-cystic mass, presumably arising from the left ovary and measuring 110 × 55 × 99 mm. The tumor contained a multilocular cystic component with thick septa delimiting fluid-filled and hemorrhagic areas, some of which exhibited fluid-blood levels, as well as a solid component showing intermediate T2 signal intensity, T1 isointensity, diffusion restriction, and heterogeneous enhancement after gadolinium administration. Twisting of the left laterouterine pedicle was also visualized adjacent to the mass. The right ovary appeared normal. MRI further confirmed mild uterine enlargement, endometrial thickening to 5 mm, and moderate intraperitoneal ascites (Figure 2).

**Figure 2:**
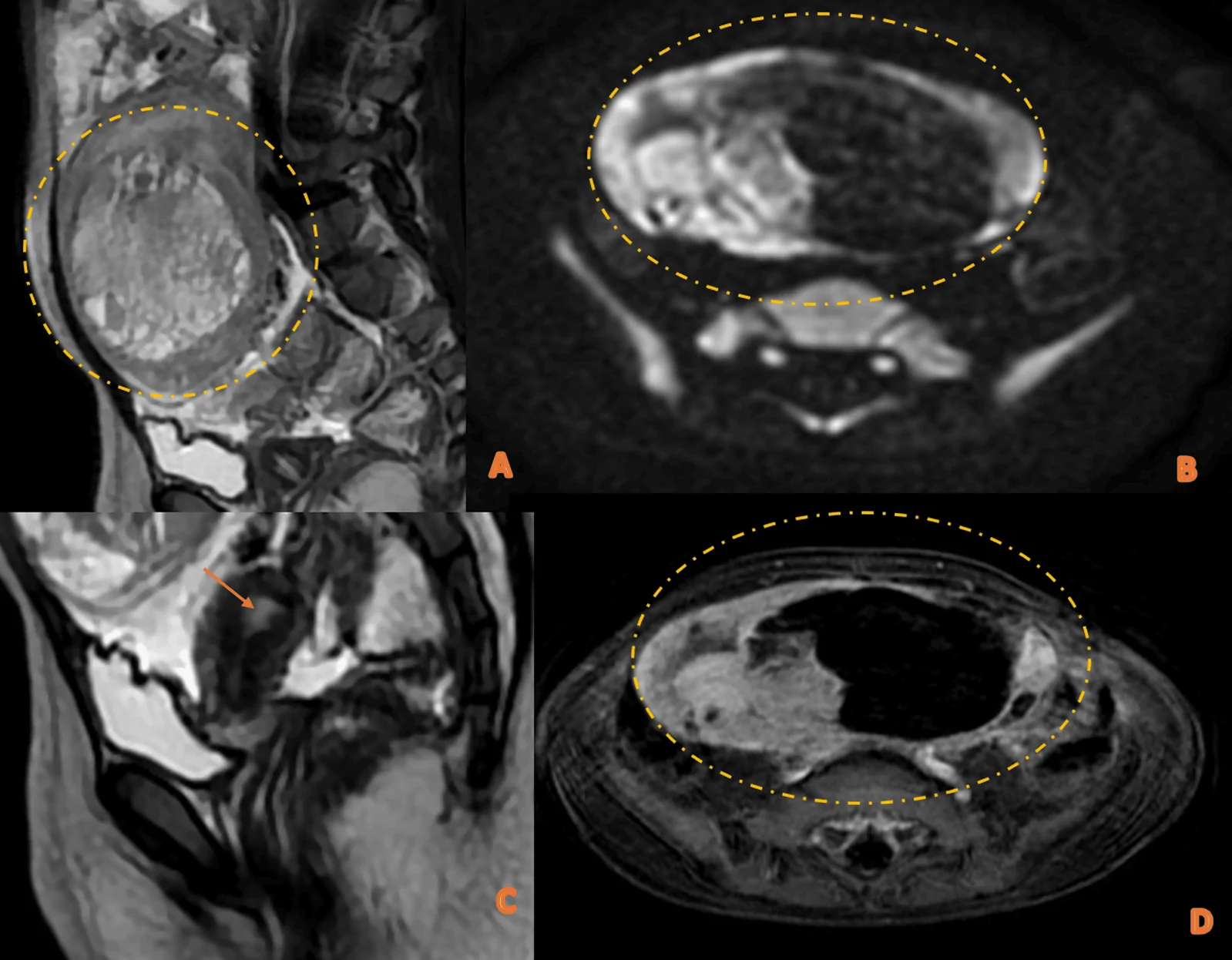
Pelvic magnetic resonance imaging Pelvic magnetic resonance imaging demonstrating a large left ovarian multilocular solid-cystic mass (yellow circle) on coronal T2-weighted (A), diffusion-weighted imaging (B), sagittal T2-weighted (C), and axial contrast-enhanced T1-weighted (D) sequences. The lesion demonstrates diffusion restriction and heterogeneous enhancement and is associated with endometrial thickening (orange arrow).

Following the initial diagnostic workup, upfront surgical management was undertaken. Complete surgical exploration revealed a well-encapsulated tumor arising from the left ovary, with no macroscopically detectable abnormalities of the peritoneal surfaces, fallopian tubes, or contralateral ovary. Ascites was present and sampled for cytological examination. A complete left oophorectomy was performed, preserving the ipsilateral fallopian tube, contralateral ovary, and uterus. An omental specimen was also obtained.

Gross pathological examination demonstrated a 10.5 × 8 × 6 cm ovarian tumor with a heterogeneous cut surface composed of solid whitish areas alternating with multilocular cystic spaces. Microscopically, the tumor exhibited a lobulated architecture composed of solid areas containing cystic spaces lined by detached tumor cells and filled with eosinophilic material. The tumor cells were round, with vesicular, nucleolated nuclei and eosinophilic, focally clear cytoplasm. Between the lobules, spindle-shaped cells were distributed within a loose, edematous, and hyalinized stroma. Foci of necrosis were also present. The mitotic index was 8 mitoses per 10 high-power fields (Figures 3, 4). 

**Figure 3:**
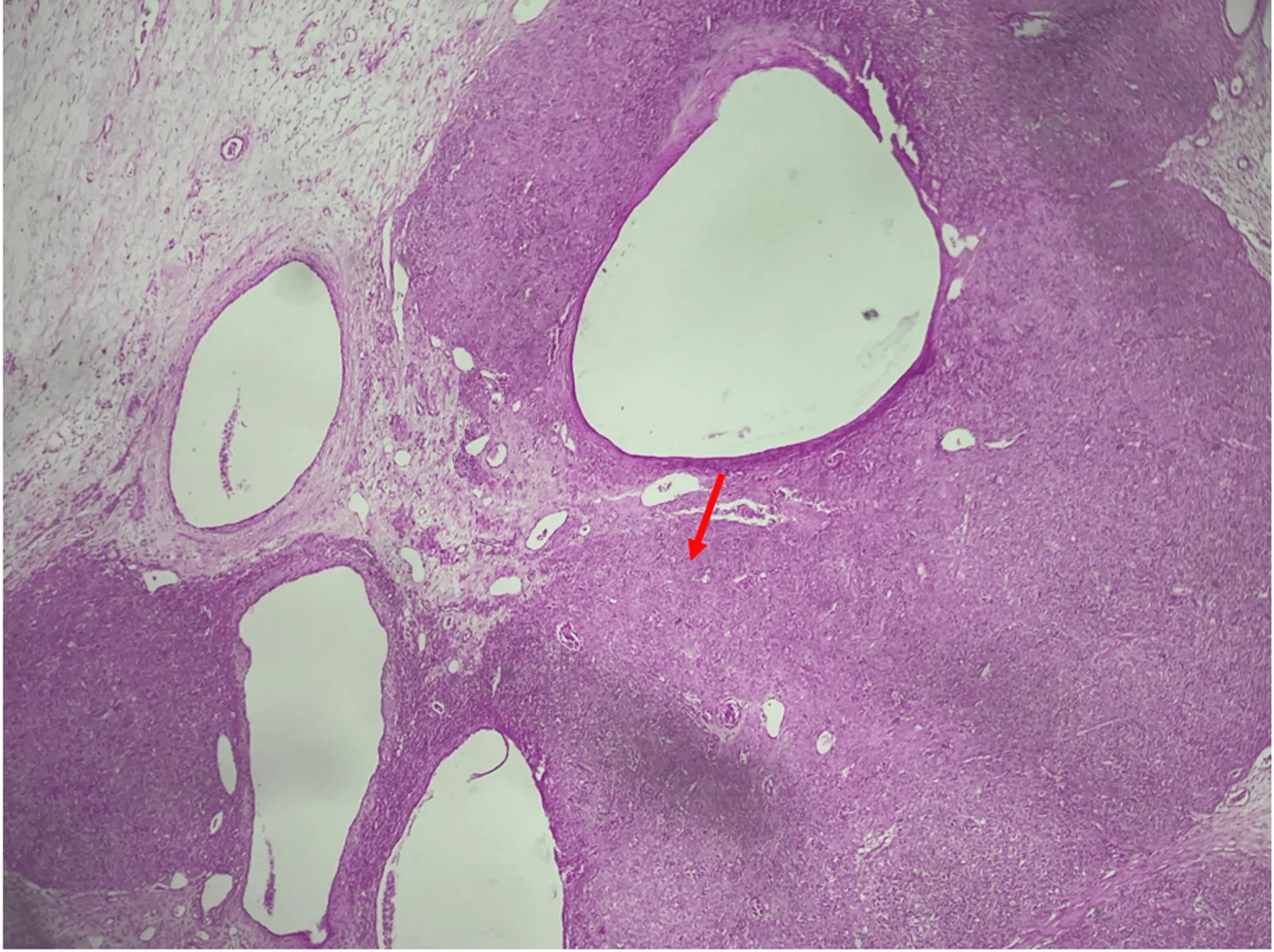
Histopathological examination Low-power photomicrograph showing a solid-cystic tumor proliferation (red arrow) (hematoxylin and eosin stain, ×40).

**Figure 4:**
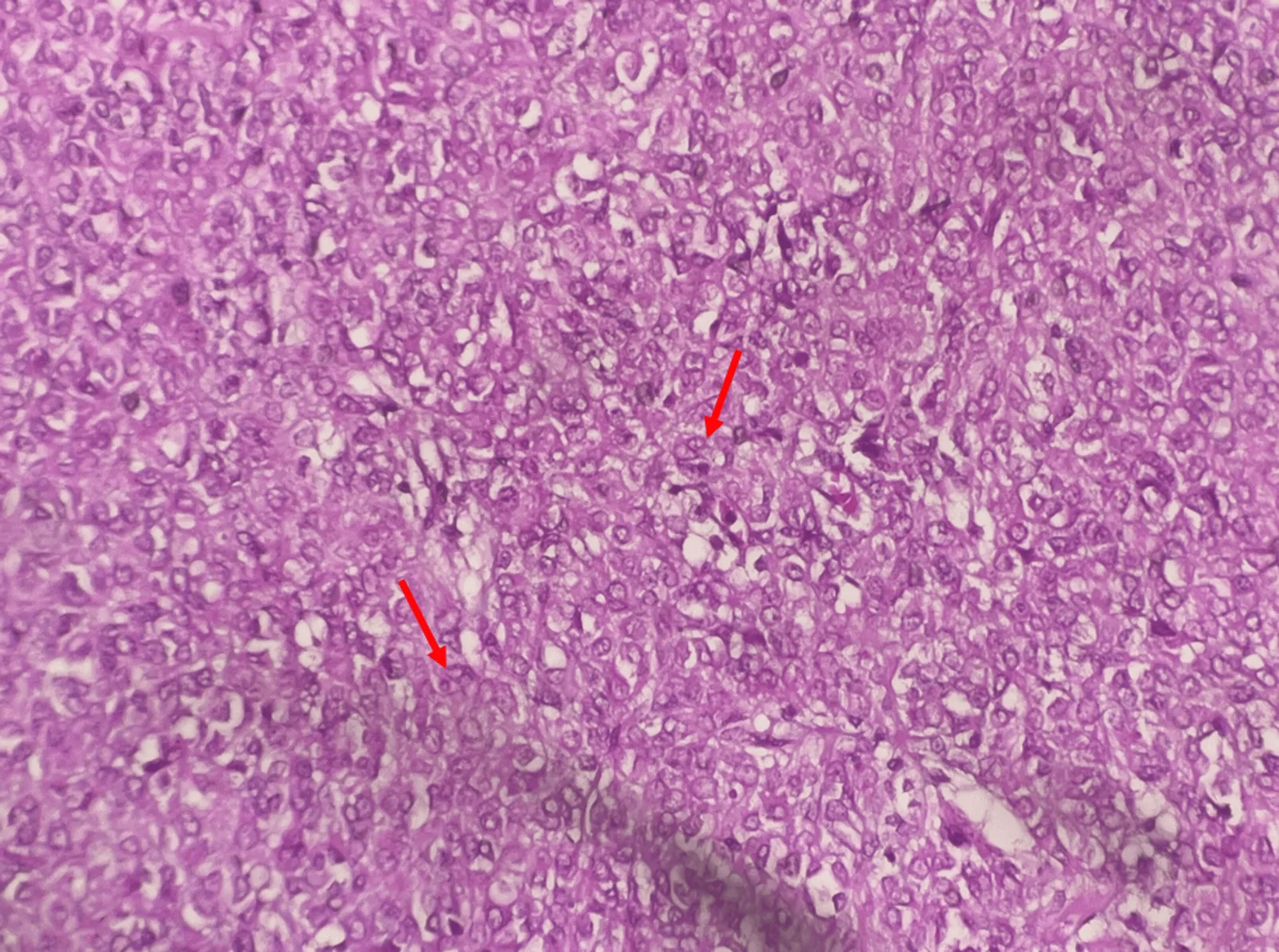
High-power histopathological examination High-power photomicrograph showing a diffuse proliferation of round to polygonal tumor cells with vesicular nuclei and pale to clear cytoplasm (red arrows) (hematoxylin and eosin stain, ×200).

Immunohistochemically, the tumor cells expressed calretinin and androgen receptor, with focal positivity for inhibin, whereas cytokeratin 19, AFP, and cluster of differentiation 117 were negative. The overall morphological and immunohistochemical findings were consistent with a JGCT. Cytological examination of the ascitic fluid was negative for malignant cells, and histological examination of the omental specimen showed no tumor involvement. Postoperative staging with abdominopelvic CT showed no residual disease (Figure 5).

**Figure 5:**
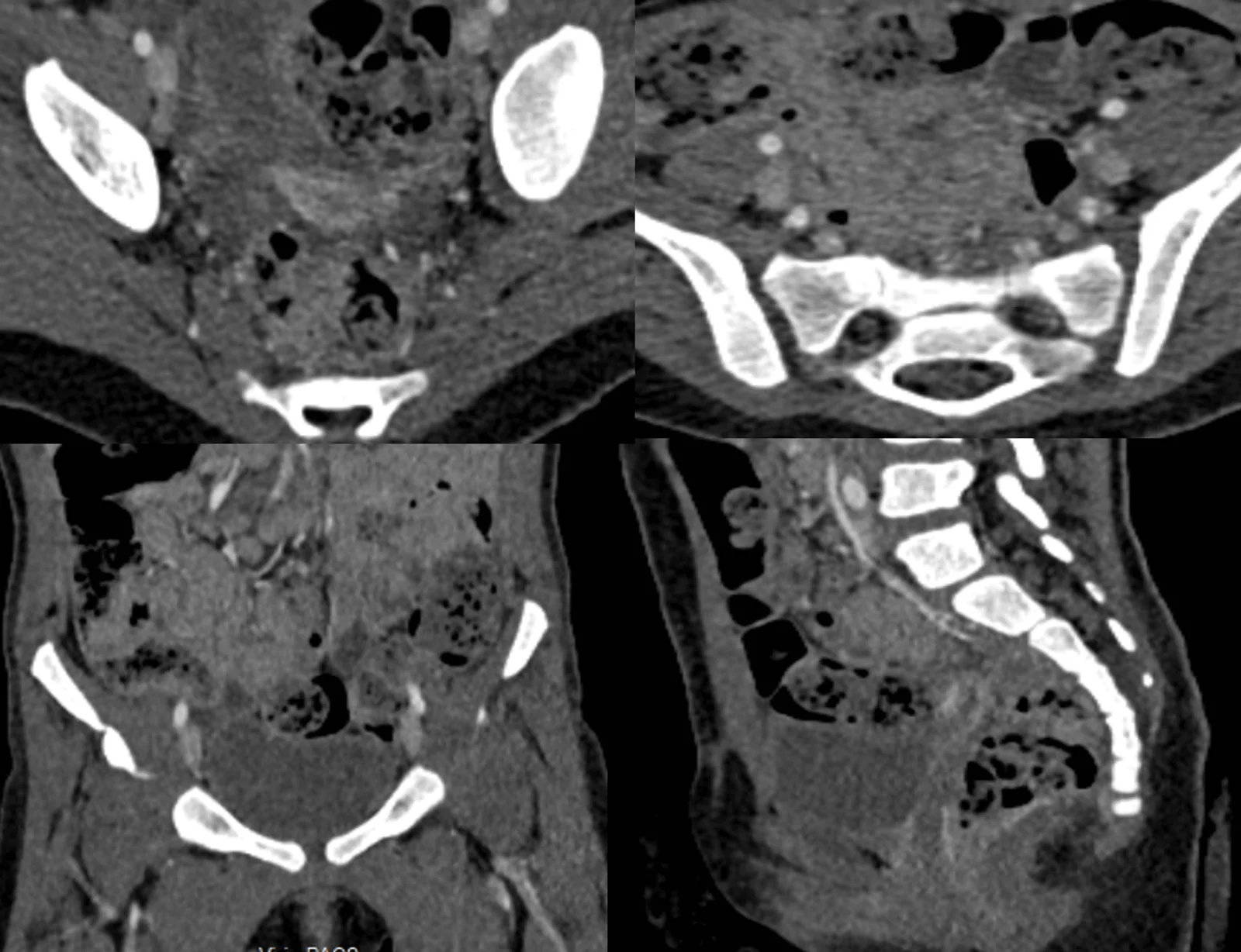
Postoperative contrast-enhanced abdominopelvic computed tomography Postoperative contrast-enhanced abdominopelvic computed tomography showing no evidence of pelvic tumor recurrence or residual pelvic mass.

Following referral to our department, the patient's clinical, radiological, surgical, and pathological findings were reviewed by our multidisciplinary tumor board. Given the complete resection of a tumor confined to the left ovary, with an intact capsule, negative ascitic fluid cytology, negative omental sampling, and no evidence of extraovarian involvement, the tumor was classified as International Federation of Gynecology and Obstetrics (FIGO) stage IA. No adjuvant chemotherapy was indicated, and the patient was managed with active surveillance. Follow-up was scheduled every three months and included clinical examination, hormonal assessment with serum inhibin B, estradiol, LH, and FSH, together with abdominopelvic ultrasonography. The previously elevated inhibin B and suppressed gonadotropin levels returned to normal, and serum estradiol, whose preoperative result was unavailable for review, was within the normal range during postoperative follow-up. All hormonal parameters remained normal thereafter. At 12 months, the estrogenic manifestations had completely resolved, and there was no clinical, biochemical, or radiological evidence of recurrence.

## Discussion

Unlike the adult subtype, JGCT predominantly affects children and young women, exhibits distinct histopathological and molecular characteristics, and is generally associated with a favorable prognosis when diagnosed at an early stage [4-6]. Nevertheless, its rarity and heterogeneous clinical presentation may complicate timely recognition, particularly in very young children, in whom endocrine manifestations often constitute the earliest clinical clue [1].

In prepubertal girls, endocrine manifestations are among the most characteristic presenting features of JGCT and result from autonomous hormone secretion by neoplastic granulosa cells [3,7]. Estrogen excess may manifest clinically as progressive breast development, leukorrhea, vaginal bleeding, accelerated linear growth, and advanced bone age, although the combination and severity of these findings vary considerably among patients [1,3,8,9]. In our patient, these manifestations evolved sequentially over several months, with unilateral breast development as the initial symptom, followed by bilateral thelarche, persistent leukorrhea, recurrent vaginal bleeding, and, ultimately, detection of a pelvic mass. This chronological progression illustrates how endocrine manifestations may precede abdominal symptoms and provide an important window for earlier diagnosis. Therefore, in toddlers presenting with progressive signs of peripheral precocious puberty, an estrogen-secreting ovarian tumor should be considered even in the absence of abdominal pain or a palpable mass [1,4]. Early pelvic imaging may shorten the diagnostic delay and facilitate detection while the disease is still confined to the ovary, when surgery alone may be curative [1,8].

Beyond these clinical manifestations, hormonal assessment provides an important diagnostic clue by supporting the peripheral origin of precocious puberty. Gonadotropin concentrations are typically suppressed through negative feedback, whereas estradiol and granulosa cell-derived biomarkers, particularly inhibin B, may be elevated [1,3,7]. In our patient, the markedly elevated inhibin B concentration (>5000 pg/mL; reference range, <182 pg/mL), together with suppressed gonadotropin levels, provided strong biochemical evidence of a hormonally active SCST. Serum estradiol had been requested during the initial evaluation, but the preoperative result was unavailable for review. Bone age assessment was not performed. Although these missing data limit the complete characterization of estrogen exposure at presentation, the clinical manifestations and hormonal profile strongly supported the functional nature of the tumor. Beyond its diagnostic value, inhibin B is also useful for postoperative surveillance, as its serum concentration may reflect tumor activity and rising levels may precede clinically or radiologically detectable recurrence [7,10].

Imaging plays a central role in the evaluation of pediatric ovarian masses by determining their organ of origin, characterizing tumor morphology, assessing disease extent, and guiding surgical planning [11]. Transabdominal ultrasonography is the recommended first-line imaging modality in children because of its accessibility and lack of ionizing radiation, whereas MRI provides superior tissue characterization and is particularly valuable for indeterminate or complex ovarian masses, local staging, and preoperative planning [11]. The radiological appearance of JGCT is highly variable and lacks pathognomonic features. These tumors may range from predominantly solid to predominantly cystic masses, although a heterogeneous solid-cystic morphology with internal hemorrhage is frequently described [2,12]. MRI may reveal enhancing solid components with restricted diffusion, multilocular cystic areas, internal septations, and hemorrhagic products of varying ages [11]. In our patient, MRI demonstrated a large multiloculated solid-cystic mass with thick septa, hemorrhagic areas containing fluid-blood levels, and a solid component showing diffusion restriction and heterogeneous contrast enhancement. These findings closely resemble the mixed solid-cystic and diffusion-restricting pattern reported by Hansen et al. [12], while the predominantly unilocular cystic presentation described by Afriliani et al. [2] further illustrates the broad imaging spectrum of JGCT. Importantly, estrogenic activity may also produce secondary imaging findings that provide valuable diagnostic clues, particularly in very young girls. Uterine enlargement and endometrial thickening have been reported in hormonally active JGCTs and may strengthen suspicion of an estrogen-secreting ovarian tumor when associated with a complex adnexal mass and clinical signs of precocious puberty [11,12]. Our patient similarly exhibited mild uterine enlargement and endometrial thickening to 5 mm, providing radiological evidence consistent with prolonged estrogenic stimulation. Nevertheless, imaging alone cannot reliably distinguish JGCT from other pediatric ovarian neoplasms, and radiological findings should be interpreted in conjunction with the patient's age, endocrine manifestations, hormonal profile, and tumor markers [2,11]. In our patient, despite the large tumor size and the presence of moderate ascites, surgical exploration revealed a well-encapsulated tumor confined to the left ovary, with normal peritoneal surfaces and contralateral adnexa, and negative ascitic fluid cytology, ultimately supporting a FIGO stage IA classification. This underscores the importance of integrating radiological, intraoperative, cytological, and pathological findings for definitive staging.

Histopathological examination remains the cornerstone of JGCT diagnosis, revealing a distinctive but heterogeneous combination of solid, follicular, and cystic architectural patterns [10]. Tumors are commonly arranged in nodules or lobules separated by fibrous or edematous stroma, with irregular follicles of variable size that may contain eosinophilic or basophilic secretions. The neoplastic cells typically have abundant eosinophilic or vacuolated cytoplasm and round to oval nuclei, while mitotic activity may be prominent, and areas of hemorrhage or necrosis may also occur [3-5]. In our patient, histopathological examination demonstrated a lobulated proliferation composed of solid areas containing cystic spaces lined by luteinized cells and filled with eosinophilic material. The tumor cells were round, with vesicular nucleolated nuclei and eosinophilic, focally clear cytoplasm, while the intervening stroma was loose, edematous, and hyalinized; foci of necrosis were also present. Histopathological review of the original slides showed a mitotic index of 8 mitoses per 10 high-power fields. Immunohistochemistry is particularly valuable for confirming sex cord-stromal differentiation, especially in morphologically challenging cases. JGCTs commonly express inhibin and calretinin, although the intensity and distribution of staining may vary [3]. In our patient, tumor cells showed calretinin positivity and focal inhibin expression, together with androgen receptor positivity, supporting the diagnosis of JGCT in the appropriate morphological and clinical context. Distinguishing JGCT from adult granulosa cell tumor is particularly important, as these tumors represent biologically distinct entities. In contrast to the adult subtype, JGCT generally lacks the characteristic longitudinally grooved “coffee-bean” nuclei and typical Call-Exner bodies and more often displays irregular follicular architecture, abundant eosinophilic or vacuolated cytoplasm, and greater mitotic activity. This distinction is further supported by molecular findings: the recurrent FOXL2 c.402C>G (p.C134W) mutation, considered the molecular hallmark of adult granulosa cell tumor, is generally absent in JGCT [3,4]. Instead, recurrent activating alterations involving AKT1, particularly internal tandem duplications affecting its pleckstrin homology domain, have been identified in a substantial subset of JGCTs, supporting activation of the PI3K/AKT pathway as a potential driver of tumorigenesis [3,4]. These histological and molecular differences reinforce the concept that juvenile and adult granulosa cell tumors should be regarded as distinct biological entities rather than age-related variants of the same disease.

Complete surgical resection is the primary treatment for ovarian JGCT, with preservation of fertility whenever oncologically feasible. In children with unilateral disease confined to the ovary, fertility-sparing surgery with preservation of the uterus and contralateral ovary is the standard approach, and unilateral oophorectomy or salpingo-oophorectomy may be performed depending on tumor extent and intraoperative findings [1,4-6]. Comprehensive surgical exploration is essential for accurate staging and should include careful inspection of the entire peritoneal cavity, contralateral ovary and adnexa, assessment of the omentum and peritoneal surfaces, and collection of peritoneal washings or ascitic fluid for cytological examination. Routine systematic lymphadenectomy is generally not recommended in the absence of suspicious lymph nodes, given the rarity of lymphatic spread in granulosa cell tumors [4,5].

The role of adjuvant chemotherapy is primarily determined by disease stage and completeness of resection. Completely resected FIGO stage IA tumors are generally managed with surgery alone, given their excellent outcomes and the absence of established benefit from adjuvant chemotherapy in this setting [1,4-6]. In contrast, systemic chemotherapy is generally considered for advanced-stage, incompletely resected, or recurrent disease, whereas its role in FIGO stage IC remains controversial and should be individualized according to the circumstances of capsular rupture, the presence of residual or extraovarian disease, and other clinical risk factors. Platinum-based regimens, particularly bleomycin, etoposide, and cisplatin, have traditionally been used when systemic treatment is indicated, although pediatric treatment strategies vary across cooperative groups and protocols [4,5,13].

In our patient, complete surgical exploration revealed a well-encapsulated tumor confined to the left ovary, with no abnormalities of the peritoneal surfaces, contralateral ovary, or fallopian tubes. Ascitic fluid cytology was negative, and histological examination of the omental specimen showed no tumor involvement. A left oophorectomy alone achieved complete tumor resection while preserving the ipsilateral fallopian tube, contralateral ovary, and uterus. The tumor was therefore classified as FIGO stage IA, and no adjuvant chemotherapy was administered, in accordance with current evidence supporting surgery alone as definitive treatment for completely resected stage IA JGCT.

Despite the excellent outcomes generally associated with completely resected early-stage JGCT, recurrence remains the main concern during follow-up. Tumor stage remains the most consistently recognized prognostic factor, with contemporary pediatric cohorts confirming excellent survival outcomes for localized disease, whereas advanced stage and incomplete resection are associated with a less favorable prognosis [4,5]. More recently, mitotic activity has emerged as a potentially important additional prognostic factor. In the international cohort reported by Harris et al., all recurrences for which baseline mitotic data were available occurred in tumors with a high mitotic index (>19 mitoses per 10 high-power fields) [5]. Similarly, Schneider et al. reported four recurrences, all involving tumors with high proliferative activity, including three initially classified as FIGO stage IA [4]. In our patient, histopathological review showed a mitotic index of 8 mitoses per 10 high-power fields, well below the high-risk thresholds identified in these studies. This finding provides additional favorable prognostic information alongside the stage IA classification. Nevertheless, these emerging data suggest that stage alone may not fully capture recurrence risk and support systematic assessment and reporting of mitotic activity in JGCT.

Long-term surveillance should combine careful clinical examination, particular attention to the reappearance of endocrine manifestations, serial measurement of tumor markers that were elevated at diagnosis, and periodic imaging [4,5]. Inhibin B is particularly useful as an individualized biomarker when initially elevated; however, it should not be used as the sole surveillance tool, since recurrent disease may occur without a concomitant rise in serum inhibin B. In the international cohort reported by Harris et al., inhibin B was elevated at diagnosis in 90% of tested patients, but among patients with recurrence and an initially elevated inhibin B level, only three of five evaluable patients showed renewed elevation at relapse [5]. Pelvic ultrasonography is particularly suitable for serial surveillance in children, allowing repeated assessment without exposure to ionizing radiation. In our patient, surveillance combined clinical examination, pelvic ultrasonography, and serial measurement of inhibin B, estradiol, LH, and FSH. At 12 months, the complete resolution of estrogenic manifestations, sustained normalization of the hormonal profile, and absence of clinical or radiological evidence of recurrence are reassuring. Nevertheless, continued long-term surveillance remains warranted given the potential for delayed recurrence.

## Conclusions

JGCT is a rare ovarian neoplasm in children, but its hormone-secreting nature offers a valuable opportunity for early recognition. Progressive estrogenic manifestations, particularly breast development, leukorrhea, and vaginal bleeding in a prepubertal girl, should prompt consideration of an ovarian tumor and timely pelvic imaging, even in the absence of abdominal symptoms. Early diagnosis is crucial, as complete surgical resection of localized disease is generally curative while preserving future reproductive potential.
